# A Dataset of 26 Candidate Gene and Pro-Inflammatory Cytokine Variants for Association Studies in Idiopathic Pulmonary Fibrosis: Frequency Distribution in Normal Czech Population

**DOI:** 10.3389/fimmu.2015.00476

**Published:** 2015-09-22

**Authors:** Amit Kishore, Veronika Žižková, Lenka Kocourková, Martin Petřek

**Affiliations:** ^1^Department of Pathological Physiology, Laboratory of Immunogenomics, Faculty of Medicine and Dentistry, Palacký University, Olomouc, Czech Republic; ^2^Institute of Molecular and Translational Medicine, Faculty of Medicine and Dentistry, Palacký University, Olomouc, Czech Republic

**Keywords:** MUC5B, cytokines, single nucleotide polymorphism, susceptibility, sequenom MassARRAY, idiopathic pulmonary fibrosis, Czech normal population, association study

## Introduction

Idiopathic pulmonary fibrosis (IPF) is a specific form of chronic, progressive fibrosing interstitial pneumonia with poor diagnosis and a median survival of 2–3 years from initial diagnosis (1, 2). The cellular inflammation drives the fibrotic response in lung and plays a major role in IPF pathogenesis (3). Inflammatory cells (majorly, type 2 alveolar epithelial cells) release TGF-β, the key mediator of pulmonary fibrosis, that regulates several profibrotic cytokines/chemokines, their receptors, receptor subunits, and growth factors inducing process of epithelial–mesenchymal transition (EMT) (3, 4). Among the pro-inflammatory and profibrotic cytokines involved in IPF pathogenesis, interleukin (IL)-1 (4), IL-1β (5), IL-4, IL-5 (6), IL-6Rα (4), angiogenic IL-8/CXCL-8 (7), IL-13, its receptor IL-13 Rα2 (8), and IL-33 (9) have been implicated in accelerated inflammation and irreversible damage to lung architecture with loss of alveolar-capillary barrier basal membrane leading to persistent fibrosis. Genes encoding these factors exhibit nucleotide variation that could affect the severity of immune/inflammatory reactions and extent of any subsequent dysregulated fibroproliferative activity in disease development. Furthermore, variants in mucin-encoding genes (10–13) and in genes for pathogen-associated molecular patterns (PAMPs) receptors of innate immunity known as toll-like receptors (TLRs) (14, 15) have been also implicated in IPF immunopathogenesis and related to rapid progression of the disease. Investigations of these “candidate” gene variant(s) e.g., in case-control association studies may, therefore, provide novel insight into underlying mechanism of IPF susceptibility/disease outcome and, further, may aid to develop novel diagnostic approaches and eventually therapeutic interventions based on genetic information (16).

A candidate gene study typically involves genotyping 5–50 single nucleotide polymorphisms (SNPs) within gene(s) for its coding and non-coding/regulatory regions (17). Irrespective of the number of tested gene variants; for a standard conductance, data collection, and transparent reporting of a genetic association study, the recommendations of STrengthening the REporting of Genetic Association studies (STREGA) and STrengthening the Reporting of OBservational studies in Epidemiology (STROBE) should be considered (16). In case-control studies, knowledge of frequency distribution of candidate gene loci/variants among normal (healthy control) population(s) is necessary and could be useful also for genetically related population(s) to determine the gene variants associated with disease and/or its clinical course.

The role of inflammatory and profibrotic mechanisms involving gene variation has been investigated in IPF and spectrum of susceptible polymorphic gene variants, including those in genes of immune reactions and signaling processes, have been recently reported from both genome-wide association studies (GWAS) and population-based case-control studies (14, 18, 19) performed mostly in US Caucasians, and also in some other ethnicities. The nominated gene variants, summarized in Table 1, are of different functions and implicate some yet-unanticipated pathways in IPF pathogenesis, including endoplasmic reticulum stress and unfolded protein response, cellular senescence, DNA-damage response, and already known Wnt–β-catenin signaling (20). The distribution of SNPs may greatly differ with populations (ethnicities), for example, a high frequency of *MUC5B* rs35705950*T allele in IPF cases is observed among European-Americans (14–34%) (21, 22), while its low frequency is characteristic for Asians, such as Chinese (3.3%) (23), Japanese (3.4%) (24), and Korean (1.0%) cohorts (11). Similarly, *MUC2* rs7934606*A allele exhibit frequency of 41% in Europeans and 1% in Asians (1000 Genomes Project Phase 3 allele frequencies). Furthermore, in context of participation of more than one gene in IPF pathogenesis, it will be important to analyze multiple susceptible gene variants. The approach of analyzing common and rare genetic factors in IPF susceptibility may provide novel insights into IPF and it could also be helpful in identifying population-specific rare variants, predominant panel of candidate gene variants for IPF risk and in understanding the basis of variable disease severity or progression among different populations.

**Table 1:** **List of candidate SNPs investigated in the study**.

S.No.	Location	Gene	SNP ID	Position	Region	Functional category	Reference
1	2q14.1	*IL-1* α	rs1800587	112,785,383	5′-flanking region	Pro-inflammatory cytokine	(25)
2	2q14.1	*IL-1* β	rs16944	112,837,290	Promoter	Pro-inflammatory cytokine	(25)
3	2q14.1	*IL-1* β	rs1143634	112,832,813	Exon (d_S_)	Pro-inflammatory cytokine	(25)
4	2p21	*PRKCE*	rs628877	45,698,441	Intron	Cellular signaling pathways	–
5	3q26	*LRRC34*	rs6793295	169,800,667	Exon (d_N_)	Protein-protein interaction	(26)
6	3q22.1	*TF*	rs1799899	133,756,968	Exon (d_N_)	Iron ion transport	–
7	4q13.3	*IL-8*	rs4073	73,740,307	Promoter	Pro-inflammatory cytokine	(18)
8	4q22.1	*FAM13A*	rs2609255	88,890,044	Intron	GTPase activator activity	(26)
9	4q35	*TLR3*	rs3775291	186,082,920	Exon (d_N_)	Innate immunity	(14)
10	5p15	*TERT*	rs2736100	1,286,401	Intron 2	Telomerase maintenance	(26)
11	5q31.1	*IL-13*	rs1800925	132,657,117	Promoter	Pro-inflammatory cytokine	(27)
12	5q31.1	*IL-4*	rs2243248	132,672,952	Promoter	Pro-inflammatory cytokine	(19)
13	5q31.1	*IL-4*	rs2243250	132,673,462	Promoter	Pro-inflammatory cytokine	(19)
14	5q31.1	*IL-4*	rs2070874	132,674,018	Promoter	Pro-inflammatory cytokine	(19)
15	6p21.2	*CDKN1A*	rs2395655	36,677,919	Promoter	Cell cycle regulation	(19)
16	6p21.2	*CDKN1A*	rs733590	36,677,426	Promoter	Cell cycle regulation	(19)
17	6p24	*DSP*	rs2076295	7,562,999	Intron	Binds intermediate filaments	(26)
18	10q24	*OBFC1*	rs11191865	103,913,084	Intron	Telomerase maintenance	(26)
19	11p15.5	*MUC2*	rs7934606	1,100,037	Intron	Mucus production in lungs	(26)
20	11p15.5	*MUC5B*	rs35705950	1,219,991	Promoter	Mucus production in lungs	(12, 21)
21	11p15.5	*TOLLIP*	rs5743890	1,304,599	Intron	Innate immunity	(21)
22	13q34	*ATP11A*	rs1278769	112,882,313	3′ UTR	Phospholipid translocation	(26)
23	16p12.1	*IL-4R* α	rs1801275	27,363,079	Exon (d_N_)	Pro-inflammatory cytokines	(19)
24	17p13.1	*TP53*	rs12951053	7,674,089	Intron	Cell cycle regulation	(28)
25	17p13.1	*TP53*	rs12602273	7,679,695	Intron	Cell cycle regulation	(28)
26	17q21	*MAPT*	rs1981997	45,979,401	Intron	Microtubule-associated tau	(26)
27	17q23.3	*ACE*	rs4277405	63,471,557	Promoter	Fibrotic pathway	(29)
28	17q23.3	*ACE*	rs4459609	63,471,587	Promoter	Fibrotic pathway	(29)
29	19p13	*DPP9*	rs12610495	4,717,660	Intron	Serine protease encoding	(26)

*ACE, angiotensin-converting enzyme; ATP11A, ATPase, class VI, type 11A; AZGP1, alpha-2-glycoprotein 1, zinc-binding; CDKN1A, cyclin-dependent kinase inhibitor 1A, the gene encoding p21; COMP, cartilage oligomeric matrix protein; DPP9, dipeptidyl-peptidase 9; DSP, desmoplakin; FAM13A, family with sequence similarity 13, member A; IL-13, interleukin-13; IL-8, interleukin-18; LRRC34, leucine rich repeat containing 34; MAPT, microtubule-associated protein tau; MUC2, mucin 2; MUC5B, mucin 5B; OBFC1, oligonucleotide/oligosaccharide-binding fold containing 1; PKCE, protein kinase C, epsilon); SFTP, surfactant protein; SPPL2C, signal peptide peptidase like 2C; TERT, telomerase reverse transcriptase; TF, transferrin); TGF-β, transforming growth factor-β); TNF-a, tumor necrosis factor-α); TLR3, Toll-like receptor; TOLLIP, toll interacting protein; TP53, tumor protein 53; d_s_, synonymous mutation; d_N_, non-synonymous mutation; UTR, untranslated region*.

No complex data have been yet reported on IPF-related variants in Slavonic populations, including Czechs. Starting our investigations of plausible multiple IPF susceptibility polymorphisms primarily in Czech and also related populations, we adopted allele-specific MALDI-TOF mass spectrometry-based SNPs genotyping assay for determination of gene variation in the relevant targets. Several IPF susceptible SNPs in genes of various functional categories were multiplexed, and in the first phase genotyped in probands from normal (healthy) Czech population using Sequenom MassARRAY platform. In the current dataset manuscript, we, besides genotyping methodology, report the genotype, allele, and phenotype (carriage rate) frequencies for plausible IPF susceptibility variants among normal population of Czech Republic of Western Slavonic (Caucasian) ancestry.

## Materials and Methods

### Characteristics of the study group

Ninety-six unrelated healthy subjects (45 males, 51 females), free of any disease as assessed by physician’s enquiry about their personal and family history, were enrolled. The mean age ±standard deviation was 34.5 ± 8.9 years and ranged from 18–57 years. All were Caucasians, and as assessed by surname and tracking personal history of Czech (Western Slavonic) ancestry living in Moravian region of the Czech Republic. All probands were informed about the purpose of the study and provided informed consent; the study was realized with the approval of the institutional ethics committee. Genomic DNA was isolated from peripheral blood leukocytes by standard salting out method (30).

A list of reference single nucleotide polymorphism database ID (rsSNP) was prepared for IPF-associated gene variants identified from available literature (GWAS and case-control studies) (Table 1). SNP genotyping using Sequenom iPLEX and MALDI-TOF-based MassARRAY platform allows analysis of upto 40 SNPs in a single well reaction. Primers were designed using Assay Design Suite v2.0 (ADS v 2.0) available under online tools of Agena Bioscience (https://www.mysequenom.com/Tools).

### Assay design, PCR amplification, and genotyping

For online genotyping assay design of multiplexed SNPs using ADS v2.0, an input list of SNP IDs (rsSNP of each target) was provided and following steps were followed: (1) the sequence for each rsSNP was retrieved from database and formatted accordingly, (2) the proximal SNPs for each rsSNP were identified from database, and (3) optimal primer areas were identified that result in a unique amplicons containing a target for the extension primer. To avoid extension primer rejection due to insufficient known bases, the proximal base was replaced with inosine. (4) PCR and extension primers were designed and checked for false priming, hairpin/dimer formation. The primer multiplexes with mass separation of analytes (alleles) were created. The PCR primer plex was prepared for PCR amplification, and extension primer mix was prepared for single base extension (SBE)-based iPLEX reaction. The SBE pool plex consists of multiplexed primers that anneal adjacent to polymorphic site for each reaction present together in the multiplexed assay pool. Thus, several individual DNA polymorphisms with their corresponding SNP sites could be analyzed in a single reaction. Due to the inverse relationship between peak intensity and extend primer mass, the extension primers in iPLEX assays were adjusted for concentration to ensure the possible equal intensity of extension primers.

For multiplexed PCR amplification and genotyping assay, protocol described in literature was followed (31). In brief, multiplexed PCR amplification was performed using 10 ng DNA template. A cleanup reaction for amplicons plex was performed with shrimp alkaline phosphatase (SAP) mix. These SAP cleaned amplicons plex were further subjected to SBE reaction with iPLEX mix containing extend primer plex. The iPLEX reaction products were treated with a cationic resin (SpectroCLEAN, Sequenom) to remove salts, such as Na^+^, K^+^, and Mg^2+^ ions. The desalted iPLEX extend amplicons were dispensed on SpectroCHIPs using MassARRAY^®^ Nanodispenser RS 1000 station and analyzed on matrix-assisted laser desorption/ionization time-of-flight mass spectrometry (MALDI-TOF MS)-based MassARRAY analyzer for target allele(s). As assay control of SNP genotyping, a duplicate, positive, and negative control samples were included in each assay plate. In each assay, the specific ddNTP incorporated at target site could be identified with peak representing increase in mass of extend primer. The assay peak spectrum and call cluster plot resulting from MALDI-TOF MS analysis were analyzed with MassARRAY Typer 4.0.20 software that traces primer masses to the assayed alleles.

### Statistical analysis

Each SNP was tested for Hardy–Weinberg Equilibrium (HWE) by Pearson’s goodness-of-fit, Chi-square (χ2) test. SNPs within HWE (*p* > 0.05) and sufficiently common (Minor Allele Frequency, MAF > 5%) in the general population were included. Phenotype frequency (carriage rate) was calculated as proportion of individuals carrying one or two copies of a particular allele on one or both (maternal or paternal) chromosomes.

## Results

Using online Assay Design Suite v2.0 for primer designing, two plexes were generated. Plexes I and II consisted of 22 and 7 SNPs, respectively. For a successful assay, inosine was used instead of proximal SNP in the extend primer sequence for rs2736100, rs2243250, and rs4277405 of plex I.

In assay control of multiplexed SNP genotyping, SNP rs2395655 in *CDKN1A* showed low assay-success rate (< 95%) and two SNPs *DSP* rs2076295 and *TOLLIP* rs5743890 were found as positive in no template control. These SNPs failed the quality control assessments and were removed from further analysis (Table 2). The genotyping assays success rates for all other analyzed SNPs were 98–100%. In our Czech healthy control population, all analyzed SNPs were in HWE, except for *IL-4R*α rs1801275 that exhibited minor deviation (*p* = 0.04) reflecting a small anomaly, so the locus was not excluded from analysis (Table 2).

**Table 2:** **Genotype distribution and phenotype frequency of candidate SNPs in normal Czech population**.

S.No.	Genetic variant	Assay rate (%)	Genotype	*n* **=** 96	HWE	Allele	Phenotype frequency
1	*IL-1* α rs1800587	100	CC CT TT	39 45 12	0.86	C T	0.88 0.59
2	*IL-1* β rs16944	100	GG GA AA	46 38 12	0.35	G A	0.88 0.52
3	*IL-1* β rs1143634	100	CC CT TT	56 33 7	0.49	C T	0.93 0.42
4	*PRKCE* rs628877	99^a^	GG GT TT	61 27 7	0.11	G T	0.93 0.36
5	*LRRC34* rs6793295	100	TT TC CC	45 45 6	0.23	T C	0.94 0.53
6	*TF* rs1799899	100	GG GA AA	81 15 0	0.41	G A	1.00 0.16
7	*IL-8* rs4073	100	AA AT TT	19 51 26	0.50	A T	0.73 0.80
8	*FAM13A* rs2609255	100	TT GT GG	53 37 6	0.89	T G	0.94 0.45
9	*TLR3* rs3775291	100	GG GA AA	41 44 11	0.88	G A	0.89 0.57
10	*TERT* rs2736100	100	TT GT GG	29 48 19	0.91	T G	0.80 0.70
11	*IL-13* rs1800925	100	CC CT TT	49 38 9	0.68	C T	0.91 0.49
12	*IL-4* rs2243248	98^b^	TT GT GG	80 14 0	0.44	T G	1.00 0.15
13	*IL-4* rs2243250	98^b^	CC CT TT	64 25 5	0.24	C T	0.95 0.32
14	*IL-4* rs2070874	100	CC CT TT	66 27 3	0.91	C T	0.97 0.31
15	*CDKN1A* rs733590	100	TT CT CC	36 47 13	0.71	T C	0.86 0.63
16	*OBFC1* rs11191865	100	GG AG AA	33 45 18	0.70	G A	0.81 0.66
17	*MUC2* rs7934606	100	GG AG AA	41 48 7	0.16	G A	0.93 0.57
18	*MUC5B* rs35705950	100	GG GT TT	80 14 2	0.16	G T	0.98 0.17
19	*ATP11A* rs1278769	100	GG GA AA	48 39 9	0.79	G A	0.91 0.50
20	*IL-4R* α rs1801275	100	AA AG GG	68 22 6	0.04	A G	0.94 0.29
21	*TP53* rs12951053	100	AA CA CC	81 15 0	0.41	A C	1.00 0.16
22	*TP53* rs12602273	100	CC GC GG	81 15 0	0.41	C G	1.00 0.16
23	*MAPT* rs1981997	100	GG GA AA	67 26 3	0.81	G A	0.97 0.30
24	*ACE* rs4277405	100	TT CT CC	36 48 12	0.51	T C	0.88 0.63
25	*ACE* rs4459609	100	AA CA CC	37 46 13	0.83	A C	0.86 0.61
26	*DPP9* rs12610495	100	AA GA GG	54 35 7	0.69	A G	0.93 0.44

*^a^One sample for rs628877*.

*^b^Two samples each for rs2243250 and rs2243248 were not genotyped*.

*HWE, Hardy–Weinberg equilibrium*.

Among analyzed SNPs in cytokines, their receptor and sub-units, *IL-4* rs2243248 exhibited highest genotype (TT = 0.85), allele frequency (T = 0.93) and carriage rate (T = 1.00). Besides cytokines, we also report allelic frequency of rs3775291 in *TLR3*, an innate immune system receptor. In present report, four out of 26 SNPs, *viz*., *TP53* rs12951053, *TP53* rs12602273, *TF* rs1799899, and *IL-4* rs2243248 showed complete absence of their respective homozygous genotype CC, GG, AA, and GG, and exhibited high phenotype frequency (1.00) for allele A, C, G, and T, respectively (Table 2). For *MUC5B* rs35705950*T risk-allele, allelic and phenotype frequencies were found as 9% and 17%, respectively.

The genotype frequency and allele frequency for the 26 analyzed SNPs are available online at ALlele FREquency Database with Sample UID: SA004336Q (http://alfred.med.yale.edu/alfred/pophetgraph.asp?sampleuid=SA004336Q&cutoff=0.25) and will be publicly available at dbSNP database with the release of dbSNP Build (B144) (http://www.ncbi.nlm.nih.gov/SNP/snp_viewTable.cgi?handle=LIGP).

## Discussion

The present dataset reports the genotype distribution, genotype, allele, and phenotype frequency of 26 gene variants involved in immune-related pathomechanisms of IPF in normal Czech population using Sequenom MassARRAY based genotyping platform. Besides the relevance to the delineation of immunogenetic component of IPF, the knowledge of frequency distribution of gene variants in normal populations is of considerable importance for their evaluation as genetic markers in susceptibility, manifestation, prognosis, and potentially treatment of diseases in different populations (32).

A SNP rs35705950 in the putative promoter of *MUC5B* has been shown to exhibit strong association with both familial interstitial pneumonia and IPF (33). The observed rs35705950*T risk-allele frequency of 9% in normal Czech population was in concordance with other reports in normal Caucasians of European-American descents, as 9–11% in American (33), 10% in UK Caucasians (34), 11% in French (22), and 4.3% in Germans (24) populations among Europeans. Interestingly, the *MUC5B* promoter polymorphism is observed less frequently in normal Asian populations, such as 0.8% in Japanese (24), 0.7% in Chinese (23), and <1% in Koreans (11). Overall, mucin glycoprotein encoding MUC5B has role in normal lung function by regulating immune function, microbial population, airway infection, and mucociliary clearance in lungs (35, 36).

Among analyzed cytokines, IL-4 has significant role in IPF pathogenesis by regulating fibroblast functions, such as chemotaxis, proliferation, collagen synthesis, myofibroblast differentiation, and T_h_1/T_h_2 equilibrium (19). The angiogenic IL-8 was shown as predictive for early stage of IPF (37) and as poor IPF survival (38). Additionally, IL-13 and IL-13 pathway markers (39) and the innate immune signaling receptor *TLR3* have been suggested as potential markers of rapidly progressive form of IPF. Several recent studies have suggested that defective TLRs are linked to dysregulated fibrogenesis and have key role in myofibroblast activation, increased profibrotic cytokines, collagen deposition, fibrosis, and tissue destruction and, thus, promoting the progression of disease during the later phase of IPF (14, 15, 40, 41).

Of the four variants that exhibited absence of homozygous genotypes in this data report: (1) the frequency of *TP53* rs12951053 CC genotype has been reported as 6% in Caucasian HC (28), 1.2% in European and Africans and relatively higher in Asian (11.9% in Han Chinese and 11.6% in Japanese) populations (http://snp-nexus.org/temp/snpnexus_10220/results.html); (2) For *TP53* rs12602273, CC genotype frequency has been reported as 3% in Caucasian healthy controls (28); (3) For *TF* rs1799899, AA genotype frequency has been reported as 0.6% in European, and 0.0% among African, Han Chinese, and Japanese populations (http://snp-nexus.org/temp/snpnexus_10168/results.html); and (4) For *IL-4* rs2243248, low GG genotype frequency (*IL-4*, -1098 G/T) has been reported in another independent study for characterization of *IL-4* gene polymorphism in a relatively small cohort of IPF patients of same ethnicity (19).

The present findings are widely applicable in IPF genetics research in other related populations as well. In a current research initiatives in immunogenetics by HLA-NET network, a working group for population definitions and sampling strategies in population genetics analyses strongly recommend the usage of geographical and/or cultural criteria (with anthropological considerations) to describe human populations instead of *a priori* misclassifications of racial and ethnic groups (42). In this context, Central Europe populations have been demonstrated as similar and genetically homogeneous (32, 43, 44). Therefore, the present findings are relevant for IPF gene case-control studies not only in Czech but also in neighboring populations, namely Slovak and Polish, and also in Germans and Austrians, as we could recently exemplify in preliminary investigations of immune-related IPF susceptible variants in Czech and German population cohorts (10, 13).

## Conclusion

The present data on a spectrum of 26 gene variants including 10 variants of immune and inflammatory response (cytokines/chemokines and TLR) and their frequency distribution in normal Czech (Western Slavonic, Caucasian) population has wider application as standard control along with cases in association studies for IPF. It is also relevant in other fibrotic lung diseases among Czech and genetically related/neighboring population(s) and in the wider context for further delineation of the role of immune and inflammatory reactions in this debilitating disease.

## Conflict of Interest Statement

The authors declare that the research was conducted in the absence of any commercial or financial relationships that could be construed as a potential conflict of interest.

## References

[1] Raghu G Collard HR Egan JJ Martinez FJ Behr J Brown KK An official ATS/ERS/JRS/ALAT statement: idiopathic pulmonary fibrosis: evidence-based guidelines for diagnosis and management. Am J Respir Crit Care Med (2011) 183:788–824. 10.1164/rccm.2009-040GL 21471066 PMC5450933

[2] Spagnolo P Sverzellati N Rossi G Cavazza A Tzouvelekis A Crestani B Idiopathic pulmonary fibrosis: an update. Ann Med (2015) 47:15–27. 10.3109/07853890.2014.982165 25613170

[3] Agostini C Gurrieri C. Chemokine/cytokine cocktail in idiopathic pulmonary fibrosis. Proc Am Thorac Soc (2006) 3:357–63. 10.1513/pats.200601-010TK 16738201

[4] Zhang J Tian XJ Zhang H Teng Y Li R Bai F TGF-beta-induced epithelial-to-mesenchymal transition proceeds through stepwise activation of multiple feedback loops. Sci Signal (2014) 7:ra91. 10.1126/scisignal.2005304 25270257

[5] Strieter RM Mehrad B. New mechanisms of pulmonary fibrosis. Chest (2009) 136:1364–70. 10.1378/chest.09-0510 19892675 PMC2773361

[6] Furuie H Yamasaki H Suga M Ando M. Altered accessory cell function of alveolar macrophages: a possible mechanism for induction of Th2 secretory profile in idiopathic pulmonary fibrosis. Eur Respir J (1997) 10:787–94. 9150314

[7] Antoniou KM Tzouvelekis A Alexandrakis MG Sfiridaki K Tsiligianni I Rachiotis G Different angiogenic activity in pulmonary sarcoidosis and idiopathic pulmonary fibrosis. Chest (2006) 130:982–8. 10.1378/chest.130.4.982 17035428

[8] Barnes JC Lumsden RV Worrell J Counihan IP O’Beirne SL Belperio JA CXCR3 is required for the IL-13 mediated upregulation of IL-13Ralpha2 in pulmonary fibroblasts. Am J Respir Cell Mol Biol (2014) 53:217–25. 10.1165/rcmb.2013-0433OC 25514189 PMC5455693

[9] Luzina IG Pickering EM Kopach P Kang PH Lockatell V Todd NW Full-length IL-33 promotes inflammation but not Th2 response in vivo in an ST2-independent fashion. J Immunol (2012) 189:403–10. 10.4049/jimmunol.1200259 22634619 PMC3381874

[10] Kishore A Zissel G Zizkova V Mueller-Quernheim J Petrek M Association of mucin (MUC2, MUC5B) gene variants with idiopathic pulmonary fibrosis (IPF) in a German population: a pilot study using MassARRAY technology. Tissue Antigens (2015) 85:340 10.1111/tan.12557

[11] Peljto AL Selman M Kim DS Murphy E Tucker L Pardo A The MUC5B promoter polymorphism is associated with IPF in a Mexican cohort but is rare among Asian ancestries. Chest (2015) 147:460–4. 10.1378/chest.14-0867 25275363 PMC4314820

[12] Peljto AL Zhang Y Fingerlin TE Ma SF Garcia JG Richards TJ Association between the MUC5B promoter polymorphism and survival in patients with idiopathic pulmonary fibrosis. JAMA (2013) 309:2232–9. 10.1164/ajrccm-conference.2015.191.1_MeetingAbstracts.A4382 23695349 PMC4545271

[13] Petrek M Kishore A Zizkova V Genetic association study for idiopathic pulmonary fibrosis in a Czech population: results from a pilot study using MassARRAY technology. Am J Respir Crit Care Med (2015) 191:A4382 10.1164/ajrccm-conference.2015.191.1_MeetingAbstracts.A4382

[14] O’Dwyer DN Armstrong ME Trujillo G Cooke G Keane MP Fallon PG The toll-like receptor 3 L412F polymorphism and disease progression in idiopathic pulmonary fibrosis. Am J Respir Crit Care Med (2013) 188:1442–50. 10.1164/rccm.201304-0760OC 24070541

[15] O’Dwyer DN Armstrong ME Kooblall M Donnelly SC. Targeting defective toll-like receptor-3 function and idiopathic pulmonary fibrosis. Expert Opin Ther Targets (2015) 19:507–14. 10.1517/14728222.2014.988706 25530171

[16] Little J Higgins JP Ioannidis JP Moher D Gagnon F von Elm E Strengthening the reporting of genetic association studies (STREGA): an extension of the STROBE statement. Eur J Epidemiol (2009) 24:37–55. 10.1007/s10654-008-9302-y 19189221 PMC2764094

[17] Balding DJ. A tutorial on statistical methods for population association studies. Nat Rev Genet (2006) 7:781–91. 10.1038/nrg1916 16983374

[18] Ahn MH Park BL Lee SH Park SW Park JS Kim DJ A promoter SNP rs4073T>A in the common allele of the interleukin 8 gene is associated with the development of idiopathic pulmonary fibrosis via the IL-8 protein enhancing mode. Respir Res (2011) 12:73. 10.1186/1465-9921-12-73 21649933 PMC3141418

[19] Vasakova M Sterclova M Matej R Olejar T Kolesar L Skibova J IL-4 polymorphisms, HRCT score and lung tissue markers in idiopathic pulmonary fibrosis. Hum Immunol (2013) 74:1346–51. 10.1016/j.humimm.2013.07.011 23911740

[20] Kropski JA Lawson WE Young LR Blackwell TS. Genetic studies provide clues on the pathogenesis of idiopathic pulmonary fibrosis. Dis Model Mech (2013) 6:9–17. 10.1242/dmm.010736 23268535 PMC3529334

[21] Noth I Zhang Y Ma SF Flores C Barber M Huang Y Genetic variants associated with idiopathic pulmonary fibrosis susceptibility and mortality: a genome-wide association study. Lancet Respir Med (2013) 1:309–17. 10.1016/S2213-2600(13)70045-6 24429156 PMC3894577

[22] Borie R Crestani B Dieude P Nunes H Allanore Y Kannengiesser C The MUC5B variant is associated with idiopathic pulmonary fibrosis but not with systemic sclerosis interstitial lung disease in the European Caucasian population. PLoS One (2013) 8:e70621. 10.1371/journal.pone.0070621 23940607 PMC3734256

[23] Wang C Zhuang Y Guo W Cao L Zhang H Xu L Mucin 5B promoter polymorphism is associated with susceptibility to interstitial lung diseases in Chinese males. PLoS One (2014) 9:e104919. 10.1371/journal.pone.0104919 25121989 PMC4133265

[24] Horimasu Y Ohshimo S Bonella F Tanaka S Ishikawa N Hattori N MUC5B promoter polymorphism in Japanese patients with idiopathic pulmonary fibrosis. Respirology (2015) 20:439–44. 10.1111/resp.12466 25581455

[25] Hutyrova B Pantelidis P Drabek J Zurkova M Kolek V Lenhart K Interleukin-1 gene cluster polymorphisms in sarcoidosis and idiopathic pulmonary fibrosis. Am J Respir Crit Care Med (2002) 165:148–51. 10.1164/ajrccm.165.2.2106004 11790645

[26] Fingerlin TE Murphy E Zhang W Peljto AL Brown KK Steele MP Genome-wide association study identifies multiple susceptibility loci for pulmonary fibrosis. Nat Genet (2013) 45:613–20. 10.1038/ng.2609 23583980 PMC3677861

[27] Ding M Sheng H Shen W Zhen J Xi L Zeng J Association of the SNP rs1800925(C/T) in the interleukin-13 gene promoter with pulmonary function in Chinese Han patients with idiopathic pulmonary fibrosis. Cell Biochem Biophys (2013) 67:905–9. 10.1007/s12013-013-9580-1 23549736

[28] Korthagen NM van Moorsel CH Barlo NP Kazemier KM Ruven HJ Grutters JC. Association between variations in cell cycle genes and idiopathic pulmonary fibrosis. PLoS One (2012) 7:e30442. 10.1371/journal.pone.0030442 22291954 PMC3264581

[29] Uh ST Kim TH Shim EY Jang AS Park SW Park JS Angiotensin-converting enzyme (ACE) gene polymorphisms are associated with idiopathic pulmonary fibrosis. Lung (2013) 191:345–51. 10.1007/s00408-013-9469-1 23657604

[30] Miller SA Dykes DD Polesky HF A simple salting out procedure for extracting DNA from human nucleated cells. Nucleic Acids Res (1988) 16:1215 10.1093/nar/16.3.1215 3344216 PMC334765

[31] Bradic M Costa J Chelo IM. Genotyping with Sequenom. Methods Mol Biol (2011) 772:193–210. 10.1007/978-1-61779-228-1_11 22065439

[32] Kubistova Z Mrazek F Tudos Z Kriegova E Ambruzova Z Mytilineos J Distribution of 22 cytokine gene polymorphisms in the healthy Czech population. Int J Immunogenet (2006) 33:261–7. 10.1111/j.1744-313X.2006.00609.x 16893389

[33] Seibold MA Wise AL Speer MC Steele MP Brown KK Loyd JE A common MUC5B promoter polymorphism and pulmonary fibrosis. N Engl J Med (2011) 364:1503–12. 10.1056/NEJMoa1013660 21506741 PMC3379886

[34] Stock CJ Sato H Fonseca C Banya WA Molyneaux PL Adamali H Mucin 5B promoter polymorphism is associated with idiopathic pulmonary fibrosis but not with development of lung fibrosis in systemic sclerosis or sarcoidosis. Thorax (2013) 68:436–41. 10.1136/thoraxjnl-2012-201786 23321605

[35] Roy MG Livraghi-Butrico A Fletcher AA McElwee MM Evans SE Boerner RM Muc5b is required for airway defence. Nature (2014) 505:412–6. 10.1038/nature12807 24317696 PMC4001806

[36] Fletcher AA Evans CM Regulation of microbial populations and immune functions by Muc5b. Ann Am Thorac Soc (2014) 11:S80–S. 10.1513/AnnalsATS.201308-263MG

[37] Sterclova M Vasakova M Metlicka M Pavlicek J Kolesar L Striz I. Angiostatic versus angiogenic chemokines in IPF and EAA. Respir Med (2009) 103:1651–6. 10.1016/j.rmed.2009.05.012 19535235

[38] Richards TJ Kaminski N Baribaud F Flavin S Brodmerkel C Horowitz D Peripheral blood proteins predict mortality in idiopathic pulmonary fibrosis. Am J Respir Crit Care Med (2012) 185:67–76. 10.1164/rccm.201101-0058OC 22016448 PMC3262037

[39] Murray LA Zhang H Oak SR Coelho AL Herath A Flaherty KR Targeting interleukin-13 with tralokinumab attenuates lung fibrosis and epithelial damage in a humanized SCID idiopathic pulmonary fibrosis model. Am J Respir Cell Mol Biol (2014) 50:985–94. 10.1165/rcmb.2013-0342OC 24325475 PMC4068948

[40] Hogaboam CM Trujillo G Martinez FJ. Aberrant innate immune sensing leads to the rapid progression of idiopathic pulmonary fibrosis. Fibrogenesis Tissue Repair (2012) 5:S3. 10.1186/1755-1536-5-S1-S3 23259678 PMC3368762

[41] Trujillo G Meneghin A Flaherty KR Sholl LM Myers JL Kazerooni EA TLR9 differentiates rapidly from slowly progressing forms of idiopathic pulmonary fibrosis. Sci Transl Med (2010) 2:57ra82. 10.1126/scitranslmed.3001510 21068441 PMC3235647

[42] Sanchez-Mazas A Vidan-Jeras B Nunes JM Fischer G Little AM Bekmane U Strategies to work with HLA data in human populations for histocompatibility, clinical transplantation, epidemiology and population genetics: HLA-NET methodological recommendations. Int J Immunogenet (2012) 39:459–72. 10.1111/j.1744-313X.2012.01113.x 22533604 PMC3533781

[43] Lundmark PE Liljedahl U Boomsma DI Mannila H Martin NG Palotie A Evaluation of HapMap data in six populations of European descent. Eur J Hum Genet (2008) 16:1142–50. 10.1038/ejhg.2008.77 18398430

[44] Heath SC Gut IG Brennan P McKay JD Bencko V Fabianova E Investigation of the fine structure of European populations with applications to disease association studies. Eur J Hum Genet (2008) 16:1413–29. 10.1038/ejhg.2008.210 19020537

